# Supplementary figures and images for: Longitudinal Phenotypes Improve Genotype Association for Hyperketonemia in Dairy Cattle
Source: Animals (Basel). 2019 Dec 1;9(12):1059. doi: 10.3390/ani9121059 (PMC6941043; doi:10.3390/ani9121059)

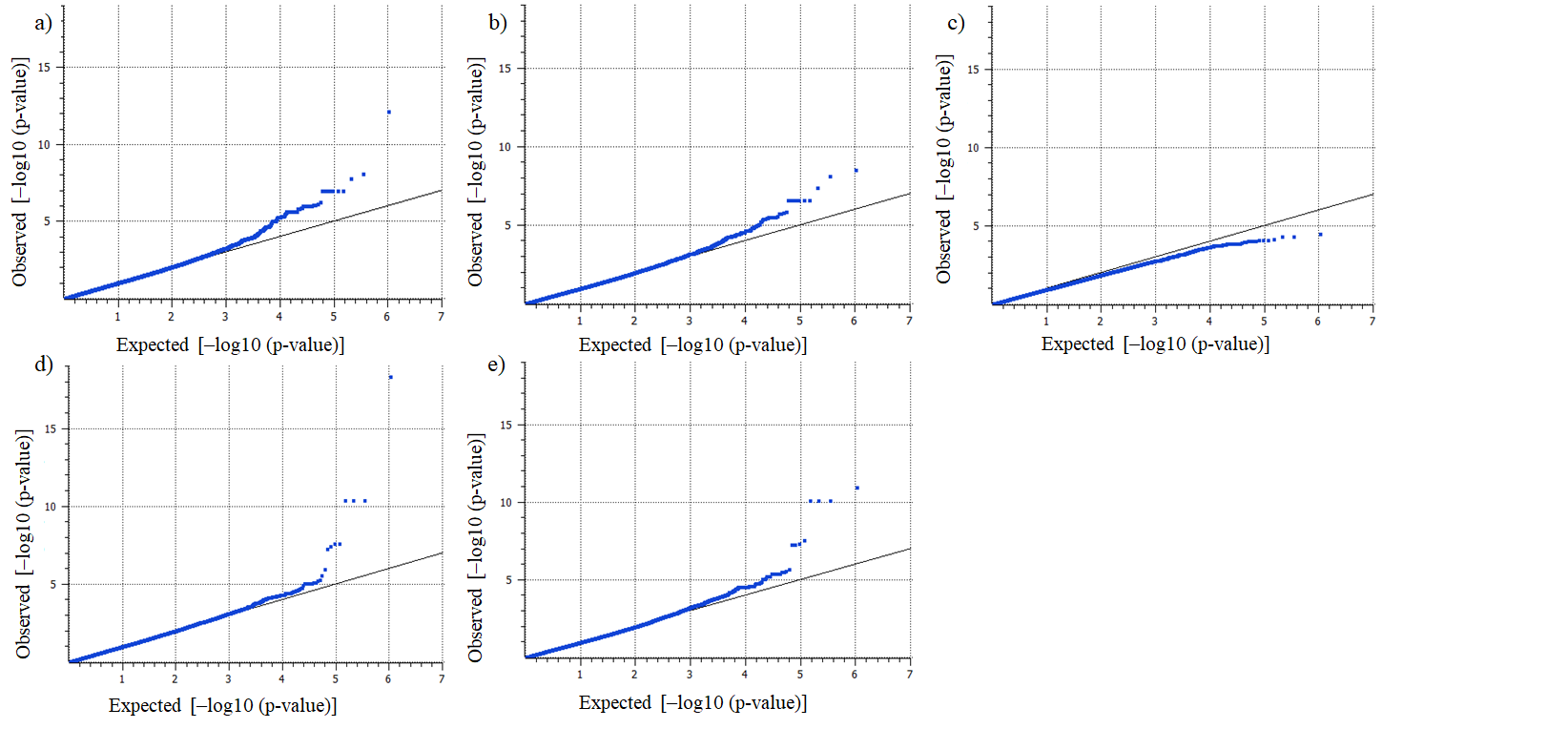

Supplement: Supplementary file 1 [file animals-09-01059-s001.zip › animals-636458-supplement-/Supporting-information-Figure-2.tiff]

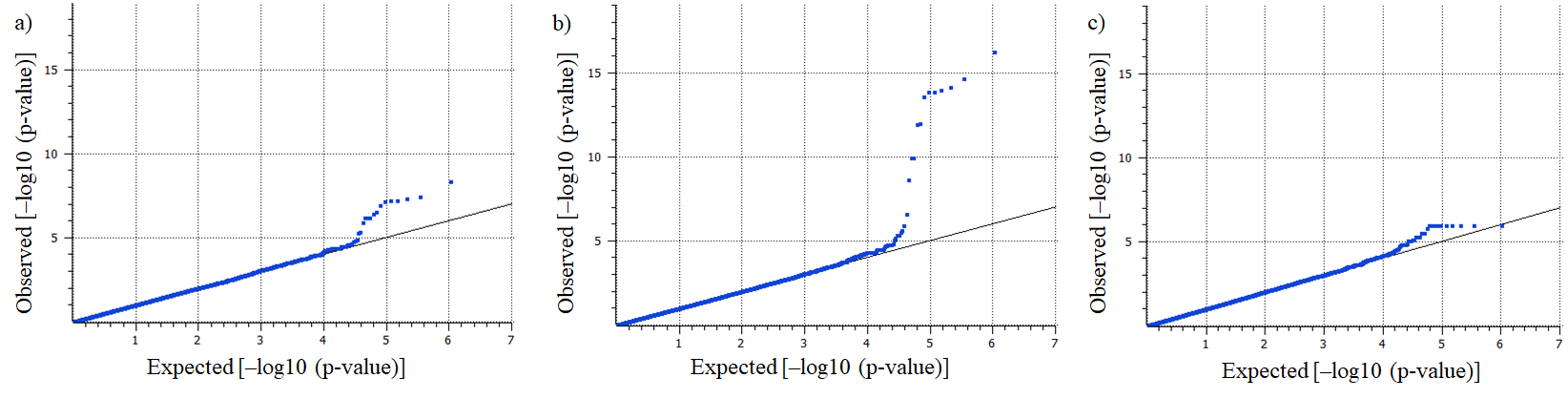

Supplement: Supplementary file 1 [file animals-09-01059-s001.zip › animals-636458-supplement-/SupportinginformationFigure1.tif]
